# Totally thoracoscopic surgical resection of left ventricular benign tumor

**DOI:** 10.1016/j.xjtc.2023.04.018

**Published:** 2023-05-29

**Authors:** Kai Xu, Zengshan Ma, Bowen Li, Zhenhua Wang, Han Song, Xiao Bai, Xiangbin Meng, Kai Liu, Xin Zhao

**Affiliations:** a Department of Cardiovascular Surgery, Qilu Hospital of Shandong University, Shandong, China; b Institute of Thoracoscopy in Cardiac Surgery, Shandong University, Shandong, China

**Keywords:** left ventricular tumor, minimally invasive cardiac surgery, thoracoscopy

## Abstract

**Objective:**

The study objective was to explore the feasibility and safety of totally endoscopic resection of a left ventricular tumor through small chest incisions without robotic assistance.

**Methods:**

Four patients with a left ventricular tumor (1 papillary fibroelastoma, 1 lipoma, and 2 myxomas) underwent surgery with peripheral cardiopulmonary bypass. The mean age of patients was 58 ± 15 years. There were 3 female patients and 1 male patient. Through 3-port incisions in the right chest, pericardiotomy, bicaval cannulation, cardiac arrest, and atriotomy, left ventricular tumor resection was performed under thoracoscopy.

**Results:**

All patients had successful resections. The cardiopulmonary bypass and aortic crossclamp times were 110 ± 14 minutes and 58 ± 19 minutes, respectively. The length of stay in the intensive care unit was 38 ± 27 hours. There were no mortalities or complications in this cohort. Patients were discharged 7 days after the operation. Transthoracic echocardiography showed that the cardiac tumor was completely removed without any residue 3 months after surgery.

**Conclusions:**

Totally endoscopic left ventricular tumor resection without a robotically assisted surgical system is feasible and reproducible. This technique could minimize surgical trauma and achieves complete tumor resection.

## Video Abstract

**Graphical abstract:**

**Figure:**
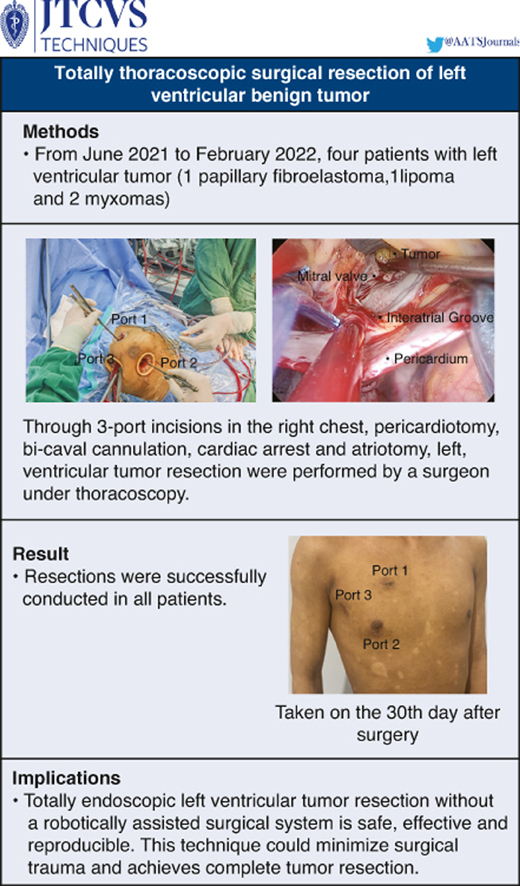

**Figure:**
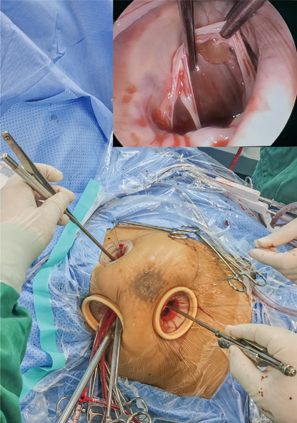
The operator's perspective of totally endoscopic resection of left ventricular tumor.

**Central Message**

Totally endoscopic left ventricular tumor resection without a robotically assisted surgical system is feasible and reproducible.

**Perspective**

The feasibility and safety of total thoracoscopic resection of ventricular tumors, especially left ventricular tumors, have not been reported. We concluded that totally endoscopic left ventricular tumor resection without a robotically assisted surgical system is feasible and reproducible. This approach may be beneficial for the visualization and thrombectomy of a left ventricular thrombus.

Cardiac tumors are a rare group of diseases. Common benign cardiac tumors include mucinous tumors, papillary fibroelastoma, rhabdomyomas, fibroma, and lipoma.^1^ Myxoma is one of the most common primary benign tumors of the heart, mostly located in the left atrium and then in the right atrium.^2^ However, the ventricular myxoma is rare, and left ventricular papillary fibroelastomas and lipomas are rarer. Minimally invasive surgical approaches have been applied to cardiac operations such as atrial septal defect repair and ventricular septal defect repair.^3^^,^^4^ It could obviously minimize surgical trauma and improve cosmetic results. In recent years, totally endoscopic techniques have been developed for atrial tumor resection.^5^ However, whether totally endoscopic left ventricular tumor resection could be achieved without a robotically assisted surgical system has not been previously investigated. In this study, we report our single-center experience with totally endoscopic resection of a left ventricular tumor through 3 small incisions in the right chest.

## Patients and Methods

### Patient Selection

This study was approved by the Institutional Review Board of Qilu Hospital of Shandong University (2016/2/25, KYLL—2016 (ks)–215). All participants provided written informed consent for publication of their study data. From June 2021 to February 2022, 4 patients with left ventricular tumors from the Department of Cardiovascular Surgery were included for this study. The inclusion and exclusion criteria are listed next.

#### Inclusion criteria

(1) The pedicle of tumor was located in the left ventricle, and the diameter of the tumor was smaller than that of the mitral annulus. (2) Pulmonary arterial systolic pressure measured by echocardiography was 60 mm Hg or less. (3) There was no history of lung disease or operations on the right chest. (4) There were no other cardiovascular diseases or chronic illnesses. (5) Preoperative chest computed tomography (CT) scan showed no diaphragmatic eventration or transverse heart position. (6) Preoperative CT scan showed no atherosclerosis of the aortoiliac tree.

#### Exclusion criteria

Patients unable to meet all of the selection criteria or unable to provide informed consent were excluded from this study. Baseline characteristics and perioperative data of the patients who underwent ventricular tumor resections are shown in Table 1. The distribution of the site of tumor occurrence (ie, the location of the pedicle) is shown in Table 2.

**Table 1:** Baseline characteristics and perioperative data of patients with left ventricular tumor

	Myxoma 1	Myxoma 2	Lipoma	Papillary fibroelastoma	Study group (n = 4)
Age (y)	37	72	65	59	58 ± 15
Gender	Female	Female	Female	Male	-
Body weight (kg)	55	42	65	57	55 ± 10
Total operation time (min)	180	225	205	175	196 ± 23
CPB (min)	106	117	126	94	111 ± 14
Aortic crossclamp time (min)	49	59	85	39	58 ± 20
Mechanical ventilation time (h)	14	8	10	9	10 ± 3
Intensive care stay (h)	16	73	17	45	38 ± 27
Postoperative hospital stay (d)	8	9	7	5	7 ± 2
Volume of chest drainage (mL)	100	150	110	80	110 ± 25
Blood transfusion (U)	2	2	2	-	3 (75%)
Postoperative analgesics	Yes	Yes	Yes	Yes	4 (100%)

*CPB*, Cardiopulmonary bypass.

**Table 2:** Distribution of the site of tumor occurrence

Pathological typology	Location
Myxoma 1	Anterior medial wall of the left ventricle
Myxoma 2	Chorda tendinea of posterior mitral leaflet
Lipoma	Anterior medial wall of the left ventricle near the apex
Papillary fibroelastoma	Posterior to the anterior mitral leaflet

### Anesthesia

After induction of general anesthesia, a left-sided double-lumen endotracheal tube was placed to allow for single-lung ventilation. The respiration rate was set between 18 and 30 breaths/min, and the arterial oxygen saturation rate was maintained at greater than 97%.

### Surgical Techniques

The patient was positioned in the supine position with the right side of the body elevated to 15° to 20°. After systemic heparinization, the right femoral artery and vein were accessed through an oblique incision along the inguinal crease. Bicaval venous drainage (26F or 28F) (Medtronic, Inc) was inserted through the right femoral vein into the inferior and superior vena cavae. The bypass circuit was completed by positioning a catheter (20F or 24F) (Medtronic, Inc) in the abdominal aorta through the right femoral artery.

**Figure 1:**
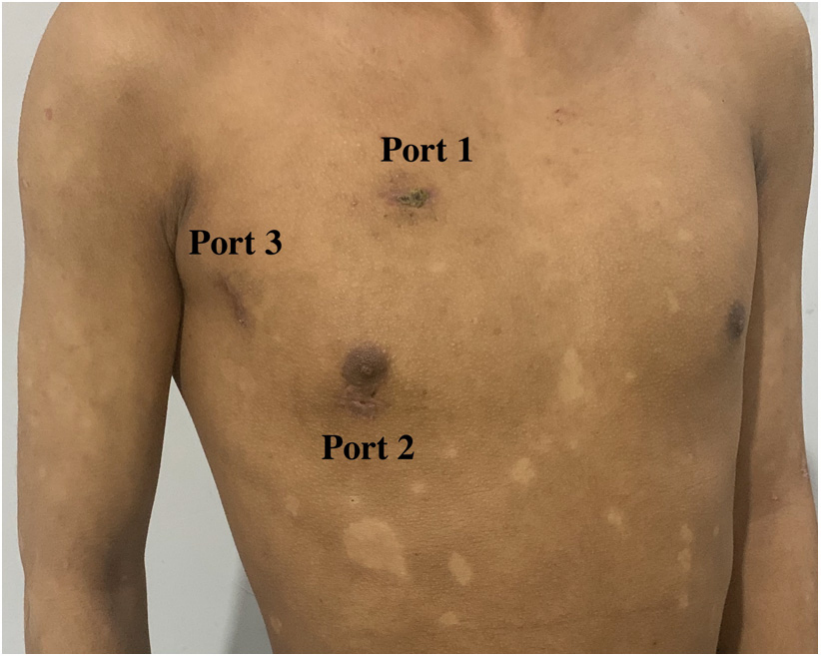
Location of the 3 ports on the right chest wall (this photograph was taken on the 30th day after surgery). Ports 1, 2, and 3 were located in the second intercostal space on the right side of the sternum, the fourth intercostal space on a midclavicular line, and the third or fourth intercostal space on the right midaxillary line, respectively.

**Figure 2:**
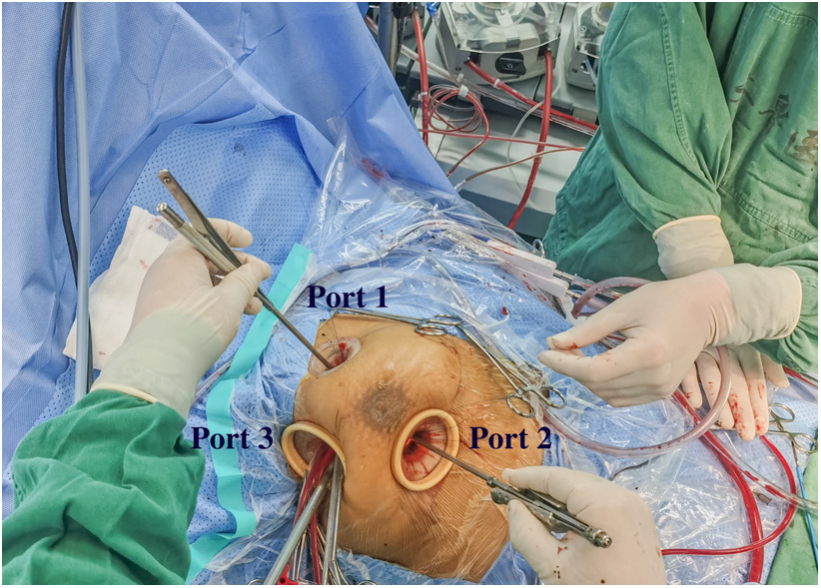
Function of the 3 ports. Port 1 was for the insertion of surgical instruments, such as tissue forceps or suture needles, using the left hand (for a right-handed operator). Port 2 was for the entry of instruments, such as scissors, handled by the right hand of the operator. Port 3 was first to be made and prepared for the placement of an endoscopic camera or thoracoscopy, CO_2_ catheter, traction line, left heart drainage tube, upper and lower chamber cannula, left heart cannula, perfusion cannula, pericardial, and atrial traction lines.

Three small incisions (ports) were made on the right side of the chest (Figure 1 and Video 1). Port 1 (1-1.5 cm) was located in the second intercostal space on the right side of the sternum (Figure 1). This port was for the insertion of surgical instruments, such as tissue forceps or suture needles, using the left hand (for a right-handed operator). Port 2 (1-1.5 cm) was for the entry of instruments, such as scissors, handled by the right hand of the operator and was located in the fourth intercostal space on a midclavicular line (Figure 1). Port 3 (1.5-2.0 cm) was located in the third or fourth intercostal space on the right midaxillary line (Figure 1). This port was first to be made and prepared for the placement of an endoscopic camera or thoracoscopy, CO_2_ catheter, traction line, left heart drainage tube, upper- and lower-chamber cannulae, left heart cannula, perfusion cannula, pericardial, and atrial traction lines (Figure 2). A plastic retractor (3 × 4 cm for ports 1 and 2, 5 × 6 cm for port 3) was inserted into each of the 3 ports to keep them open and to facilitate the insertion or withdrawal of the instruments or thoracoscopy (Figures 1 and 2).

**Figure 3:**
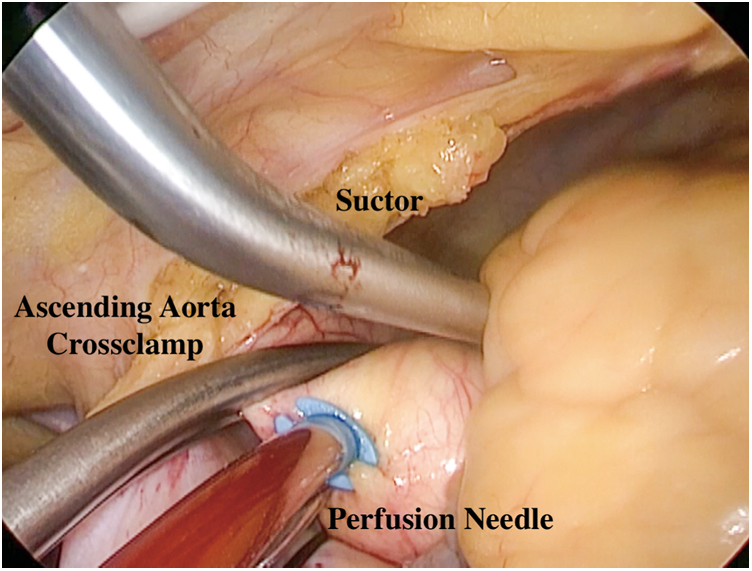
Ascending aortic crossclamp. After CPB initiation, an aortic crossclamp was positioned on the ascending aorta. A needle was inserted to the aortic root for the delivery of cold cardioplegic solution to achieve cardiac arrest.

**Figure 4:**
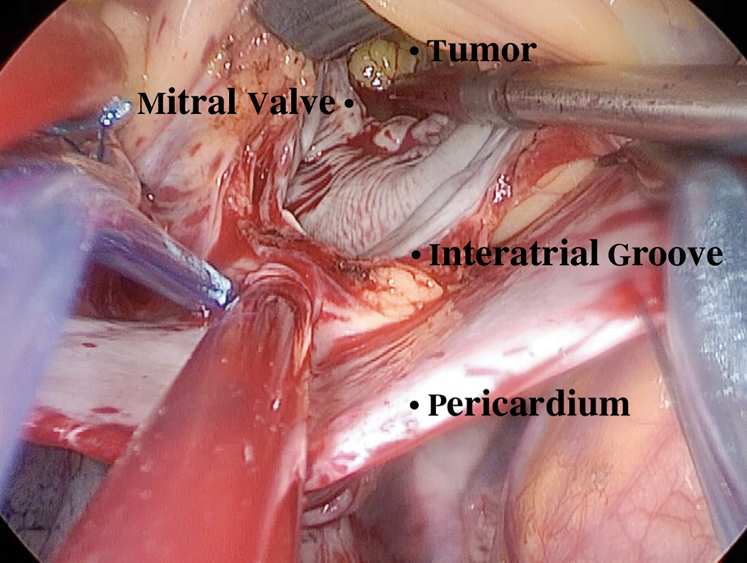
The exposure of the operating field. Pericardiotomy near the mediastinum was performed and 3 to 4 sutures were placed to suspend the bottom half of pericardium only. The left atrium was opened from a site under the interatrial groove and parallel to the atrioventricular annulus, and 4 stay sutures were placed on the incision to expose the intra-atrial structure. Through the opening mitral valve, the tumor was removed completely.

**Figure E1:**
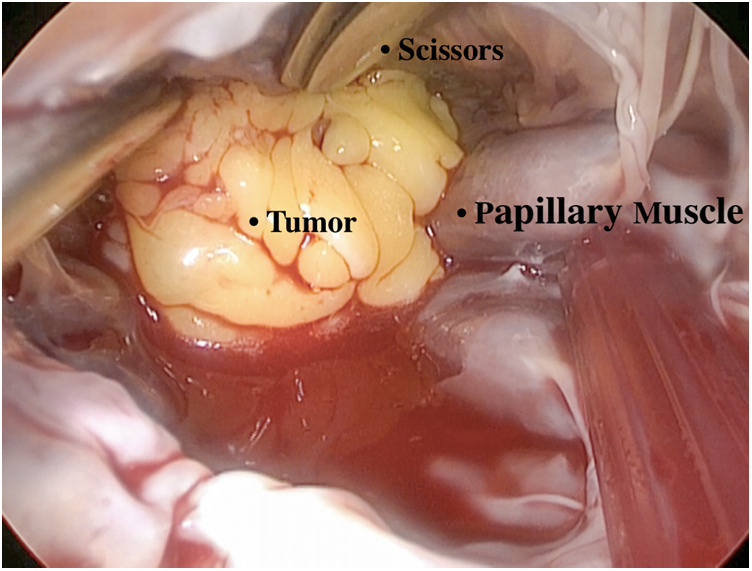
Tumor resection. This 4 × 2.5 × 2-cm lipoma was located in the anterior medial wall of the left ventricle near the apex.

**Figure E2:**
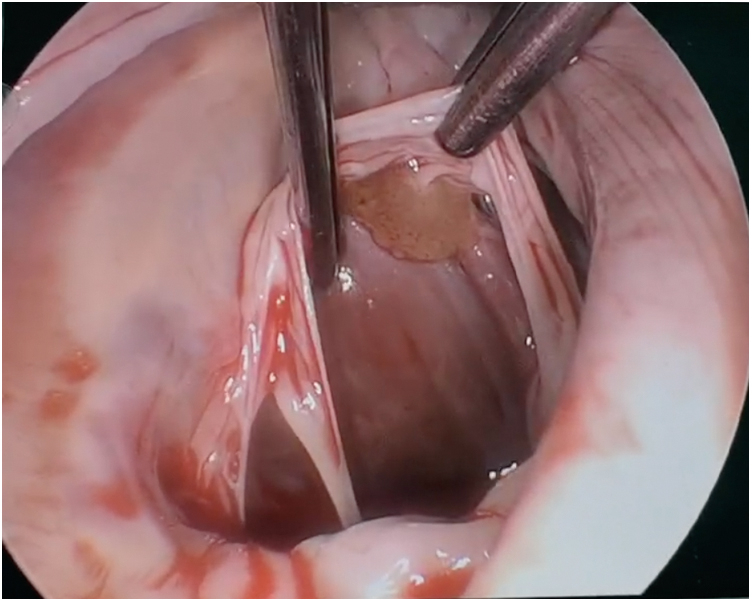
Tumor resection. This 3.5 × 3 × 1.4-cm myxoma was located in the chorda tendinea of posterior mitral leaflet.

Once the 3 ports were secured, unilateral left lung ventilation started simultaneously, and a pericardiotomy near the mediastinum was performed. Then, 3 to 4 sutures were placed to suspend the bottom half of pericardium only (Video 1 and Video Abstract). Ready-to-use caval snares were placed in the superior and inferior vena cavae before installing total cardiopulmonary bypass (CPB). After CPB initiation and cooling to 32 °C, the thoracoscopy was placed through port 2 to visualize the root of the aorta. An aortic crossclamp was positioned on the ascending aorta (Figure 3). A needle was inserted through port 3 to the aortic root for the delivery of cold cardioplegic solution to achieve cardiac arrest (Figure 3). The thoracoscopy was repositioned through port 3 to visualize the interatrial groove (interatrial sulcus). A tissue forceps and a scissors were entered through ports 1 and 2, respectively. The left atrium was opened from a site under the interatrial groove and parallel to the atrioventricular annulus, and 4 stay sutures were placed on the incision to expose the intra-atrial structure. Through the opening mitral valve, the tumor tip was excised and the tumor was removed completely (Figure 4, Figures E1 and E2, and Video 1). The left ventricle and left atrium were explored for residual tumor, and the left ventricle was filled with water to determine whether the mitral valve was well closed. The atrial sulcus incision was sutured. After evacuating the residual gas in the heart chambers, the aortic crossclamp was released. The patient was rewarmed and weaned from CPB. Autonomic resuscitation or external electrode defibrillation was performed after rewarming. Finally, extracorporeal circulation was withdrawn. The resection of the tumor was confirmed by transesophageal echocardiography. Protamine sulfate (1:1) was administered to counteract the actions of heparin. After adequate hemostasis was achieved, all instruments were removed from the chest, and a chest tube (24F) was inserted in the right pleural space through port 2 for drainage after removal of the cannulas and reconstruction of the right femoral artery and femoral vein.

### Perioperative Management

Before surgical intervention, education and counseling were provided to all participants on surgical techniques, possible clinical outcomes, potential complications, and postoperative self-care measures. Preoperative CT is necessary to exclude relative or absolute contraindications to thoracoscopic cardiac surgery such as history of previous surgery on the right lung, adhesions, diaphragmatic eventration, transverse heart position, and poor cardiac function. Lung function tests were routinely performed on all patients before the operation. The lungs were inflated every 20 minutes during the operation. After the operation, patients were monitored in the intensive care unit (ICU) overnight and received low-frequency, high-volume mechanical ventilation with a peak end-expiratory pressure of 3 to 5 cm H_2_O. Bedside chest radiographic analysis was routinely performed in the ICU to exclude complications in the lungs. Furosemide (1 mg/kg administered intravenously once daily) and methylprednisolone (0.5 mg/kg administered intravenously once daily) were used in all patients to prevent pulmonary edema. Mechanical ventilation was ceased once the patient's hemodynamics, arterial blood gas results, and spontaneous respiration stabilized. Patients were encouraged to perform respiratory exercises and have regular coughs. Routine postoperative care was provided when patients were transferred from the ICU, and patients were encouraged to get out of bed early.

### Statistical Analysis

SPSS 23 software (SPSS, Inc) was used for statistical analysis. Quantitative variables are expressed as mean ± standard deviation.

## Results

**Figure 5:**
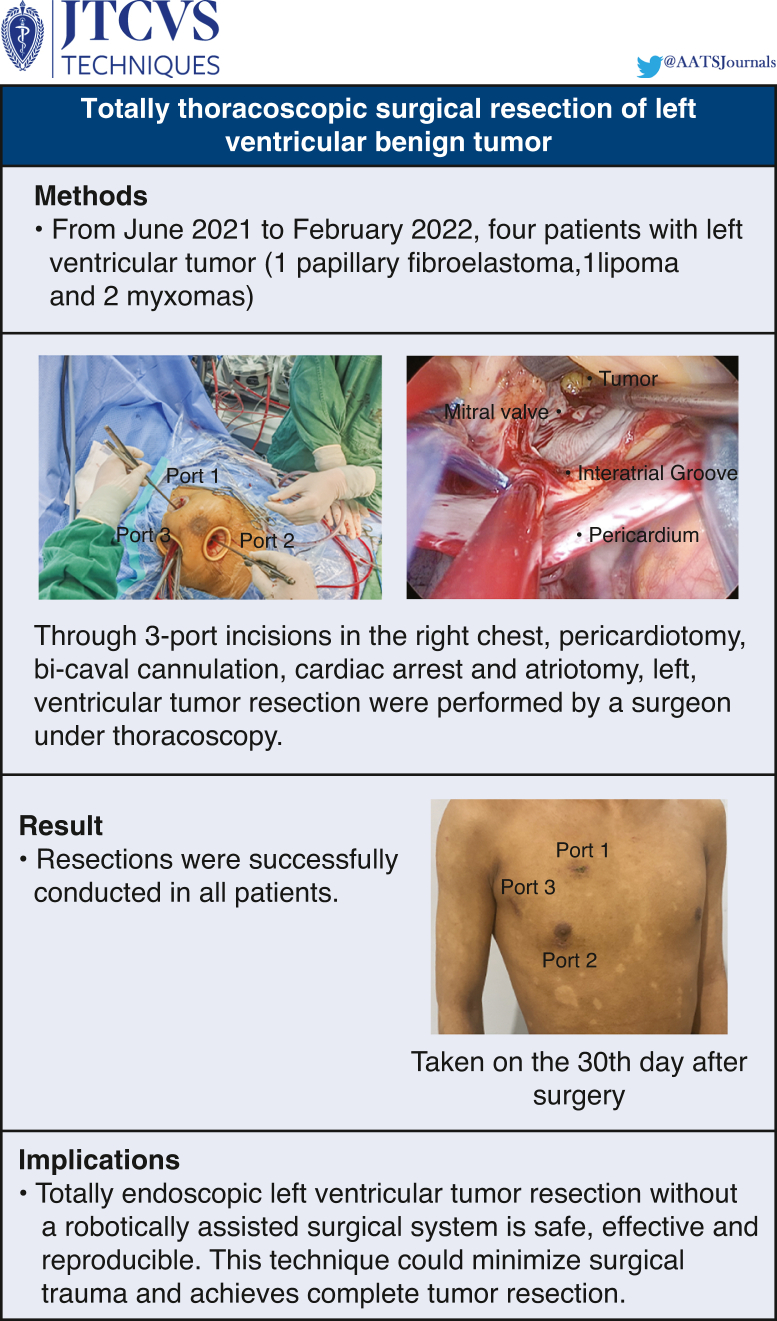
Methods, Results, and Implications arranged from top to bottom.

Resections were successfully conducted in all patients. The total operation time was 196 ± 23 minutes. The CPB and aortic crossclamp times were 106 ± 12 minutes and 49 ± 10 minutes, respectively. The length of stay in the ICU was 38 ± 27 hours. There were no mortalities and no major complications such as bleeding, stroke, renal failure, and wound infection in this cohort. Patients were discharged from the hospital in an average of 7 days after the operation. Transesophageal echocardiography immediately after the operation and 30 days after operation showed that the cardiac tumor was completely removed without any residue (Figure 5).

## Discussion

Cardiac tumors are a rare condition of the heart. As the disease progresses, some benign tumors grow into the chambers causing obstruction of blood flow and heart valve dysfunction, and can lead to acute embolism or even sudden cardiac death if the tumor is partially or completely dislodged.^6^ These serious complications may endanger the patient's life. Myxoma is the most common benign tumor of the heart, with an incidence of 0.5 per 1 million people in the population. Myxoma can grow in any chamber of the heart, but 70% to 90% of mucinous tumors grow in the left atrium.^7^ Left ventricular myxoma is rare, and papillary fibroelastoma and lipoma growing in the left ventricle are rarer. Papillary fibroelastoma and lipoma account for 8.3% and less than 10% of primary cardiac tumors, respectively.^8^ Although rare, tumors growing in the left ventricle are more prone to fragmentation and embolic complications than those growing in other cardiac chambers because of the high amplitude of left ventricular motion.^9^ Therefore, once a left ventricular tumor is diagnosed, clinical intervention must be carried out immediately.

Surgeries are the most common and effective way to treat cardiac tumors. However, with the development of medical technology and the concept of minimally invasive surgery, conventional sternotomy has been no longer overwhelmingly dominant. Small incision approach, total endoscope techniques, and even the da Vinci surgical robot can be used to achieve perfect radical treatment for certain diseases in cardiac surgery.^10^, ^11^, ^12^, ^13^

Total thoracoscopic cardiac surgery such as thoracoscopic closure of ventricular septal defect and atrial septal defect has been performed for many years.^3^^,^^14^ In recent years, it has been extended to many other intracardiac procedures such as the correction of anomalous pulmonary venous connection, atrial tumor resection, coronary artery bypass, and valve replacement or valvuloplasty.^15^, ^16^, ^17^, ^18^ The technique of total thoracoscopic resection of atrial tumors has been well established and totally thoracoscopic surgery for atrial myxomas has been performed in more than 40 patients in our department.^5^ Furthermore, robotic-assisted excision of left and right ventricular myxomas has been documented in recent years.^19^, ^20^, ^21^ However, the reproducibility and safety of total thoracoscopic resection of ventricular tumors, especially left ventricular tumors, have not been reported.

In this study, we reported 4 cases of total thoracoscopic-assisted left ventricular tumor resection, including 1 case of lipoma, 1 case of papillary fibroelastoma, and 2 cases of myxoma. In all patients, complete resections of the tumor were achieved without serious complications such as death or embolism. A critical factor in the successful surgical treatment of cardiac tumor was optimized exposure of the operating field. To fully expose the tumor for surgical manipulation, we chose to cut through the interatrial groove, with all instruments entering the left ventricle through the fully exposed mitral orifice. One of the advantages of thoracoscopy is that it could allow a near panoramic view of the left ventricle through the mitral valve orifice. By doing this, the anatomic position and morphology of the tumor could be visualized more clearly. The ventricular tissue near the apex of the heart could be clearly determined, thus allowing the operator to locate the origin of the tumor for precise excision. On the other hand, this approach may be beneficial for the visualization of left ventricular thrombus. Patients with a myocardial infarction are at high risk of developing an intracardiac appendage thrombi at the left ventricular apex.^22^^,^^23^ These thrombi might be partially resolved by thrombolytic therapy, but they are also at risk of dislodging and cause strokes. In addition, in some patients thrombi mechanically adhered to the ventricular wall and could not be dissolved easily, thus becoming a secondary cause of fresh thrombosis. Furthermore, poor cardiopulmonary function and percutaneous coronary intervention already performed might make traditional sternotomy not effective enough.^24^ For these patients, total thoracoscopic-assisted left ventricular thrombectomy might be a new therapeutic approach worth trying.

We have summarized the following technical points:^1^ For details on the patient selection, see the “Materials and Methods” section.^2^ Choose the optimal intercostal space that can facilitate visualizing the interatrial groove more clearly by checking the preoperative CT or cardiac ultrasound.^3^ Try to dissect the tumor from its pedicle completely and never crush it to avoid embolism event.^4^ After resecting the tumor, a pocket made by rubber glove or condom was used to cover the tumor, tied closely, and then removed through the chest wall incision.^5^ Take care to check the mitral valve to avoid missing the regurgitation due to chorda tendinea injury.^6^ Repeatedly flush heart cavity several times before suturing.^7^ Release CO_2_ slowly in the intraoperative field to prevent air embolism after aortic opening.

### Study Limitations

First, minimally invasive cardiac endoscopic surgery could be conducted only in a few hospitals because of technical requirements. Moreover, most surgeons in general have not been trained sufficiently in the more complex minimally invasive cardiac endoscopic procedures. Thus, total thoracoscopic cardiac surgeries have not been commonly performed or have become the standard procedure. It has been reported that minimally invasive procedures increased the duration of CPB due to the learning curve and technical complexity.^25^ Second, only 4 cases were included in this study because cases of left ventricular tumors are rare.

## Conclusions

We have preliminarily shown that total thoracoscopic surgical resection is feasible and reproducible, and we have achieved complete ventricular tumor resection successfully with this procedure.

### Conflict of Interest Statement

The authors reported no conflicts of interest.

The *Journal* policy requires editors and reviewers to disclose conflicts of interest and to decline handling or reviewing manuscripts for which they may have a conflict of interest. The editors and reviewers of this article have no conflicts of interest.

## Supplementary Data

Video 1 The operative procedure at 2 × speed. The patient was positioned in the supine position with the right side of the body elevated to 15° to 20°. Through 3-port incisions in the right chest, pericardiotomy, bicaval cannulation, cardiac arrest, and atriotomy, the left ventricular tumor was removed completely. Video available at: https://www.jtcvs.org/article/S2666-2507(23)00179-7/fulltext.

Video Abstract Four patients with left ventricular tumor underwent surgery with peripheral cardiopulmonary bypass. Through 3-port incisions in the right chest, pericardiotomy, bi-caval cannulation, cardiac arrest and atriotomy, left ventricular tumor resection were performed by a surgeon under thoracoscopy. Video available at: https://www.jtcvs.org/article/S2666-2507(23)00179-7/fulltext.
