# Supplementary figures and images for: Totally thoracoscopic surgical resection of left ventricular benign tumor
Source: JTCVS Tech. 2023 May 29;20:116–22. doi: 10.1016/j.xjtc.2023.04.018 (PMC10405254; doi:10.1016/j.xjtc.2023.04.018)

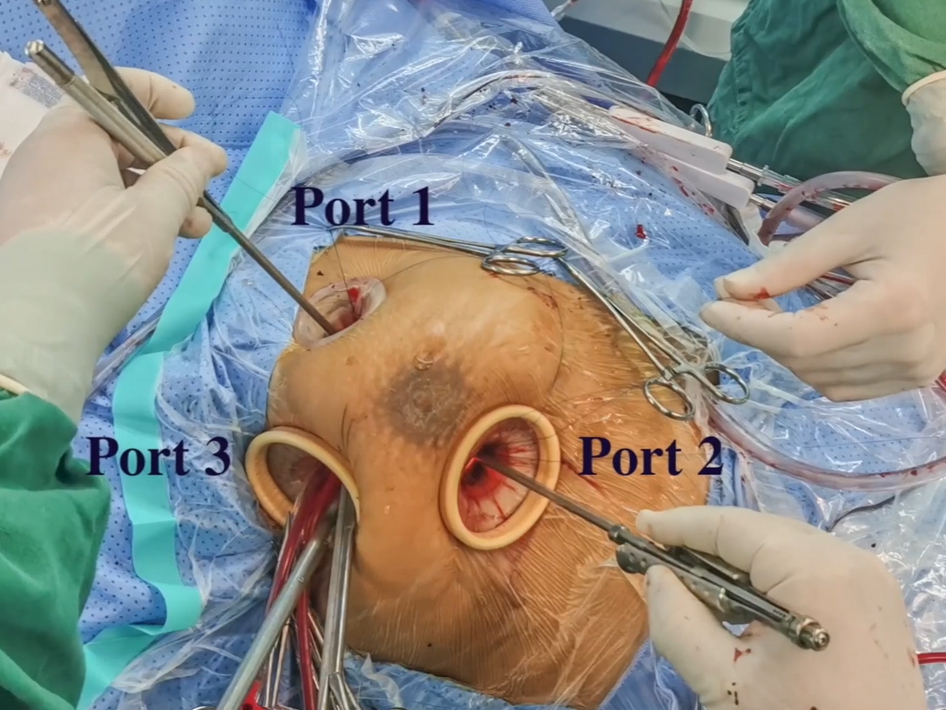

Supplement: Video 1 — The operative procedure at 2 × speed. The patient was positioned in the supine position with the right side of the body elevated to 15° to 20°. Through 3-port incisions in the right chest, pericardiotomy, bicaval cannulation, cardiac arrest, and atriotomy, the left ventricular tumor was removed completely. Video available at: https://www.jtcvs.org/article/S2666-2507(23)00179-7/fulltext. [file fx3.jpg]
